# Supplementary figures and images for: Molecular Phylogeny of Cimicoidea (Heteroptera: Cimicomorpha) Revisited: Increased Taxon Sampling Reveals Evolution of Traumatic Insemination and Paragenitalia
Source: Insects. 2023 Mar 8;14(3):267. doi: 10.3390/insects14030267 (PMC10051671; doi:10.3390/insects14030267)

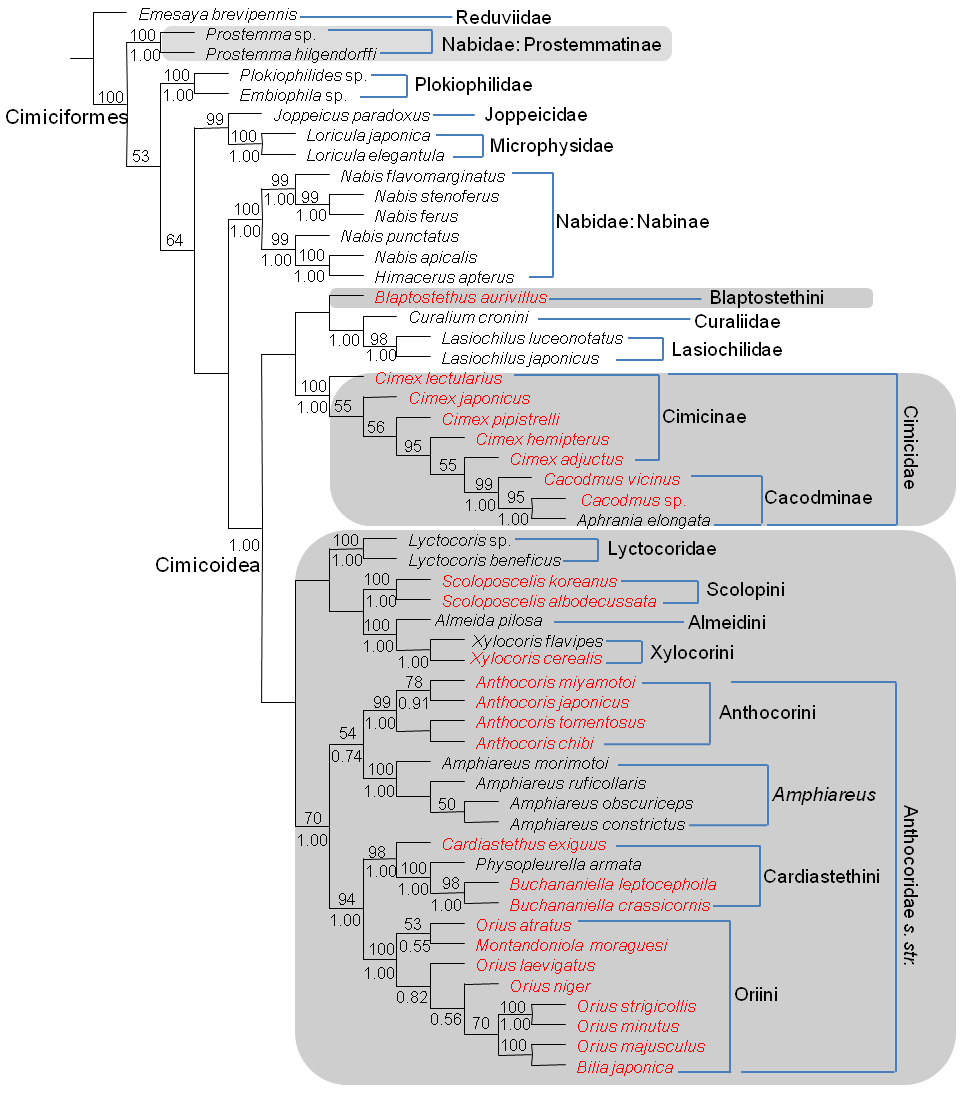

Supplement: Supplementary file 1 [file insects-14-00267-s001.zip › Supporting Information 1.tif]

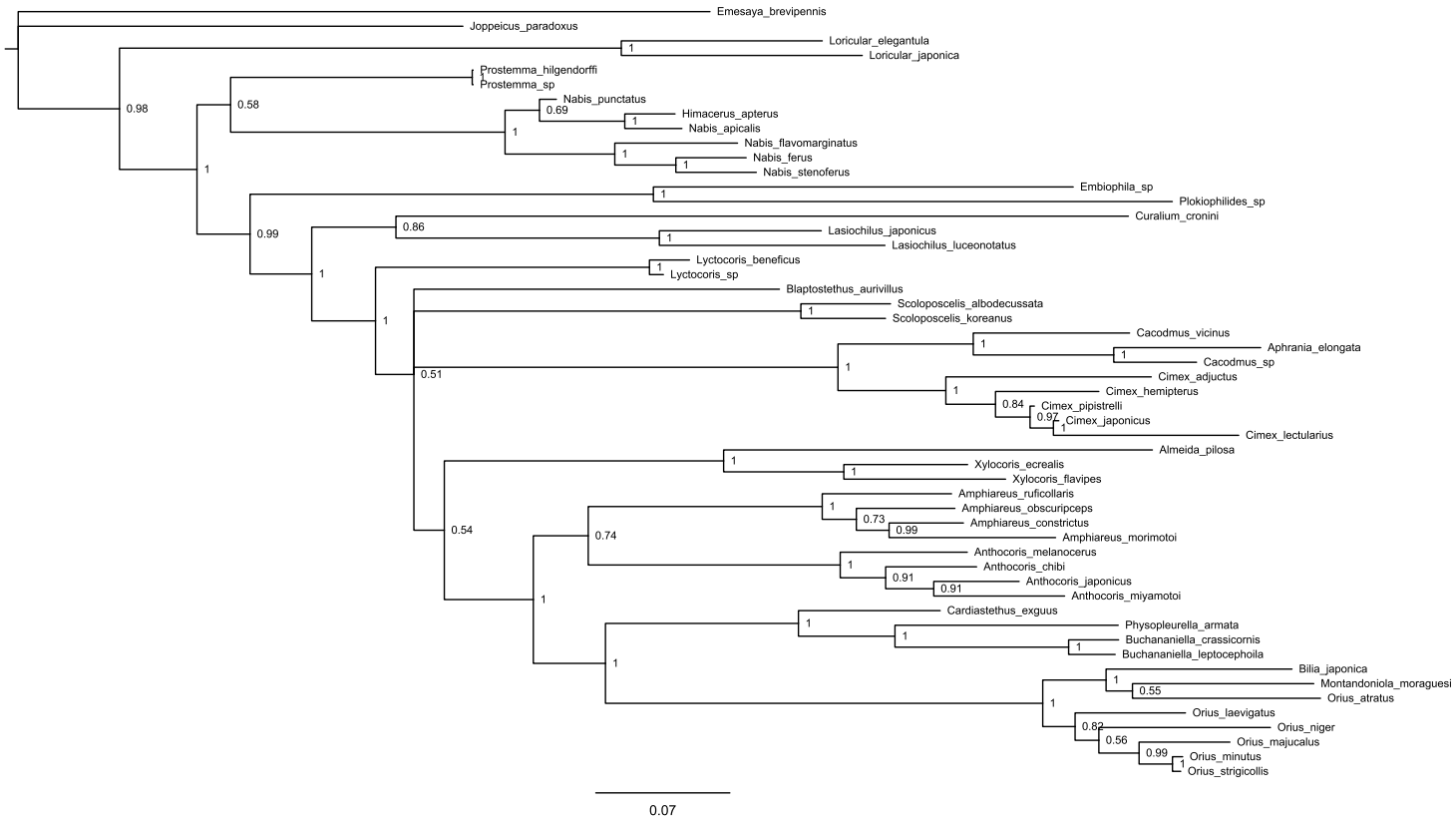

Supplement: Supplementary file 1 [file insects-14-00267-s001.zip › Supporting Information 2.pdf]

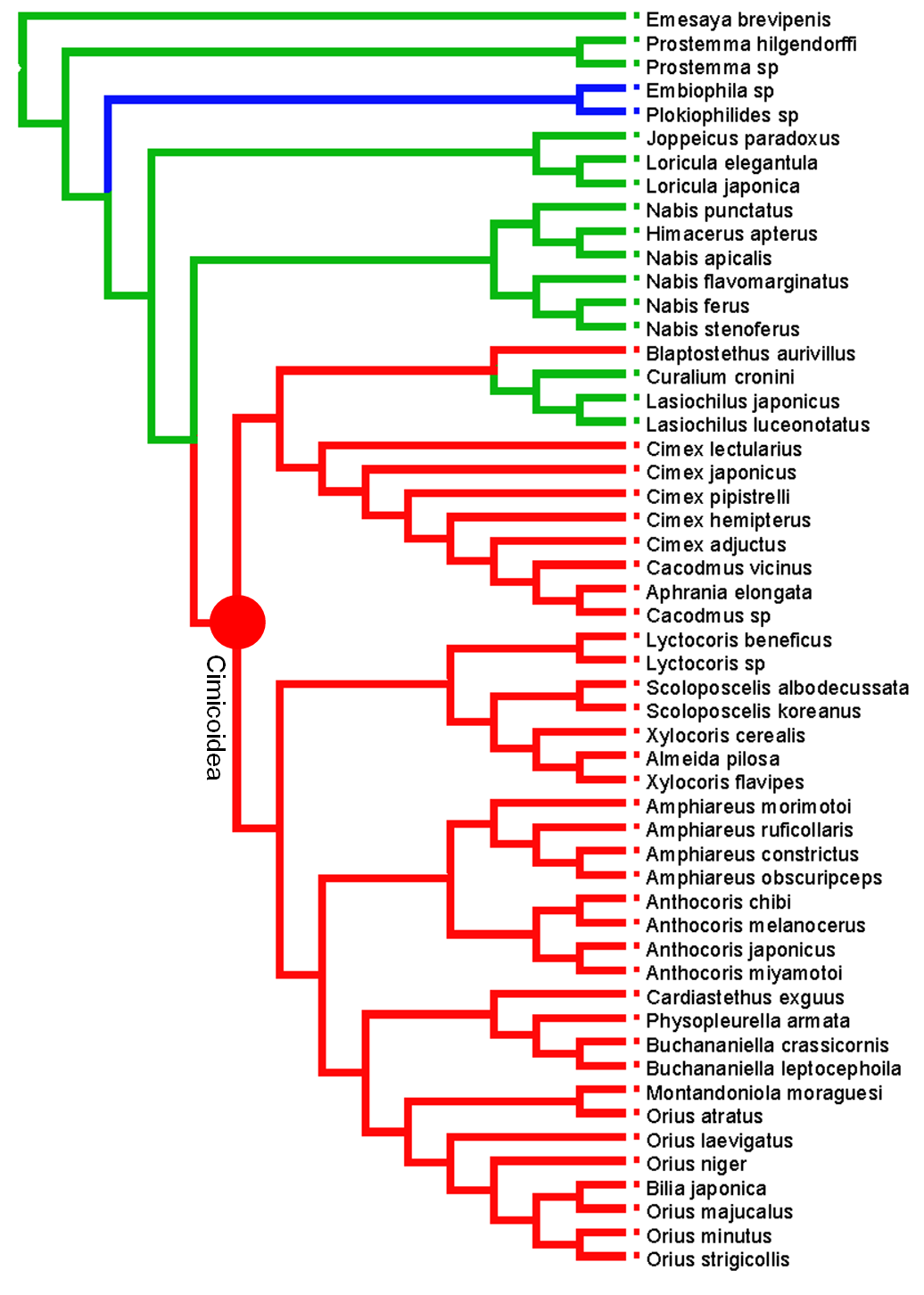

Supplement: Supplementary file 1 [file insects-14-00267-s001.zip › Supporting Information 3.tif]
